# Influence of cell wall polymers and their modifying enzymes during plant–aphid interactions

**DOI:** 10.1093/jxb/erz550

**Published:** 2019-12-12

**Authors:** Christian Silva-Sanzana, José M Estevez, Francisca Blanco-Herrera

**Affiliations:** 1 Centro de Biotecnología Vegetal, Facultad de Ciencias de la Vida, Universidad Andres Bello, Santiago, Chile; 2 Fundación Instituto Leloir and IIBBA-CONICET, Buenos Aires, Argentina; 3 Millennium Institute for Integrative Biology (IBio), Santiago, Chile; 4 Center of Applied Ecology and Sustainability (CAPES), Chile; 5 University of Edinburgh, Edinburgh, UK

**Keywords:** Aphid, cell wall, callose, cellulose, damage-associated molecular pattern (DAMP), hemicellulose, homogalacturonan, methanol, oligogalacturonides

## Abstract

Aphids are a major issue for commercial crops. These pests drain phloem nutrients and transmit ~50% of the known insect-borne viral diseases. During aphid feeding, trophic structures called stylets advance toward the phloem intercellularly, disrupting cell wall polymers. It is thought that cell wall-modifying enzymes (CWMEs) present in aphid saliva facilitate stylet penetration through this intercellular polymer network. Additionally, different studies have demonstrated that host settling preference, feeding behavior, and colony performance of aphids are influenced by modulating the CWME expression levels in host plants. CWMEs have been described as critical defensive elements for plants, but also as a key virulence factor for plant pathogens. However, whether CWMEs are elements of the plant defense mechanisms or the aphid infestation process remains unclear. Therefore, in order to better consider the function of CWMEs and cell wall-derived damage-associated molecular patterns (DAMPs) during plant–aphid interactions, the present review integrates different hypotheses, perspectives, and experimental evidence in the field of plant–aphid interactions and discusses similarities to other well-characterized models such as the fungi–plant pathosystems from the host and the attacker perspectives.

## Teaser

This review highlights the different hypotheses, perspectives, and evidence that point to cell wall polymers and modifying enzymes as relevant plant traits for aphid resistance/susceptibility.

## Introduction

At present, the consensus model of plant cell wall architecture consists of cellulose microfibrils anchored to the cell membrane, cross-linked by hemicelluloses, and embedded in a matrix of pectic polymers (Ridley *et al.*, 2001; Wolf *et al.*, 2012). In some specialized tissues such as tracheary elements and fibers in the xylem, aromatic polymers such as lignin are present in high quantities, and (glyco)proteins are also a minor but relevant plant cell wall component in all cell types (Burton *et al.*, 2010). The current model also proposes that the interaction of cellulose and hemicellulose provides stiffness to the cell wall, while the status of pectins regulates the rheological properties of the intercellular domain and cell–cell adhesion (Willats *et al.*, 2001; Wolf *et al.*, 2009; Ochoa-Villareal *et al*., 2012).

Besides regulating the mechanical and physical properties of the extracellular matrix, this polymer network represents the first defensive barrier which must be overcome by plant pathogenic agents. As a constitutive defense element of plants, cell wall polymer complexity has driven pathogens to evolve a wide range of cell wall-modifying enzymes (CWMEs) which allow them to break down these carbon-rich polysaccharide networks, gain access to the cytosolic content, and hence colonize the host. However, as a reciprocal evolutionary process, plants have developed mechanisms to monitor and adjust the composition, abundance, and distribution of the interacting polymers and its modifying enzymes to protect cell wall integrity under biotic stress circumstances. Since the feeding strategy of aphids is based on a trophic structure (stylet) that penetrates the cell wall and injects CWMEs in salivary secretions, several questions have arisen in the field of understanding the feeding behavior of aphids. These include: what is the significance of these enzymes and of the target polymers during the plant–aphid interaction and could CWMEs in aphid saliva facilitate the penetration of stylets through the extracellular matrix or can penetration be explained simply by mechanical motion? Therefore, by integrating different hypotheses, points of views, and experimental evidence in the field of plant–aphid interactions, and by comparison with other well-studied models (e.g. fungi–plant interaction), here we review and discuss the influence of cell wall polymers and CWMEs during plant–aphid interactions (Table 1).

**Table 1.** Phenotypes produced by aphid infestation in cell wall polymers and influence of CWMEs on aphid performance

Aphid	Host	Cell wall element	*Interaction phenotype*	Reference
*Myzus persicae*	Arabidopsis, *cev1* mutants	Cellulose synthase 3 (*CESA3*)	Lower population growth	Ellis *et al.* (2002)
*Diuraphis noxia*	Wheat	Callose	Callose deposits in sieve plates, plasmodesmata of companion cells and stylet tracks	Botha *et al.* (2004)
*Myzus persicae*	Arabidopsis, WT Col-0	Pectin methylesterase inhibitor 13 (PMEI13)	PMEI13 trascript is up-regulated specifically upon aphid infestation	De Vos *et al.* (2005)
*Myzus persicae*	Arabidopsis, *xth33* mutants	Endotransglucosylase/Hydrolase 33 (*XTH33*)	Aphids preffers to settle on *xth33* mutants	Divol *et al.* (2007)
*Rhopalosiphum padi*, *Myzus persicae*, *Myzus cerasi*, *Diuraphis noxia*	Barley	Callose	Callose deposits in sieve plates and plasmodesmata pores of companion cells	Saheed *et al.* (2009); Escudero-Martinez *et al.* (2017)
*Myzus persicae*	Tobacco plants overexpressing PMEs derived from *Arabidopsis thaliana* and *Aspergillus niger*	Pectin methylesterases	PME-overexpressing plants showed higher methanol emission, reducing the aphid population up to 99%	Dixit *et al.* (2013)
*Myzus persicae*	*Nicotiana tabacum*	Xyloglucan	Aphid infestation reduces the abundance of galactosylated xyloglucans	Rasool *et al.* (2017)
*Myzus persicae*	Arabidopsis, *pmei13* mutants	Pectin methylesterase inhibitor 13 (*PMEI13*)	Aphids perform longer phloem ingestions and prefer to settle on *pmei13* mutants	Silva-Sanzana *et al.* (2019)
*Myzus persicae*	Arabidopsis, WT Col-0	Homogalacturonan	Aphid infestation induce an increase in PME and PL activities, abundance of de-methylesterified HG and methanol emissions	Silva-Sanzana *et al.* (2019)

## Aphid feeding strategy

Aphids are a global threat due to the nutrient losses caused by phloem drainage, which significantly decreases crop yields (Östman *et al.*, 2003; Dedryver *et al.*, 2010). Additionally, viruses transmitted by aphids are the most relevant risk factor for the target crop. Indeed, aphids function as vectors for ~50% of the 700 known insect-borne viruses (Hooks and Fereres, 2006; Dedryver *et al.*, 2010).

**Fig. 1.**
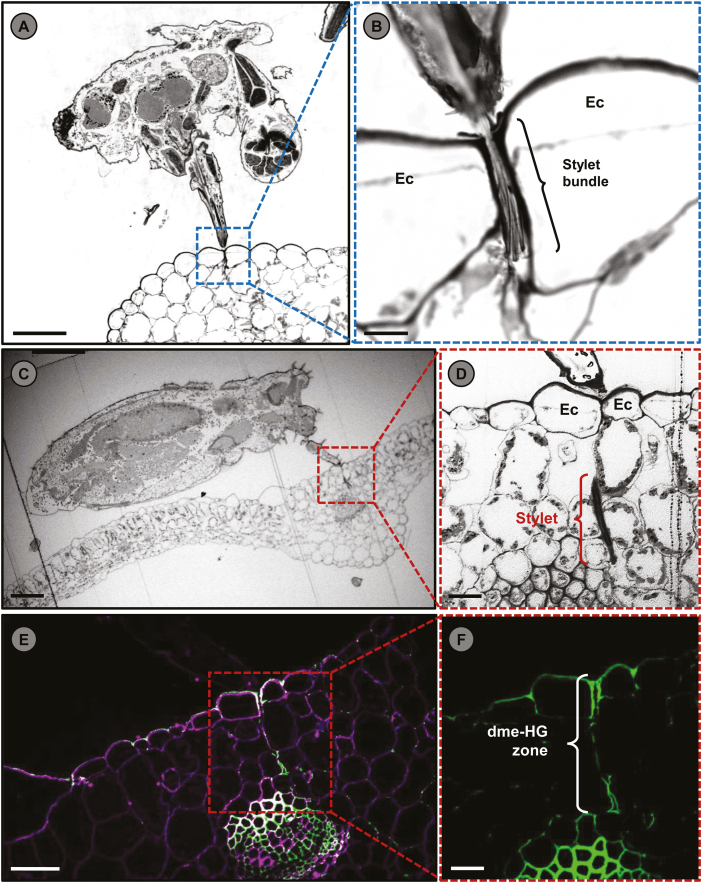
Stylet penetration through the cell wall matrix induces changes on its constituent polymers. (A) Transversal section of the head and mouthpart of an adult *Myzus persicae* aphid feeding on an Arabidopsis leaf. Scale bar=100 µm. (B) Close up of (A) showing the stylet bundle of *M. persicae* penetrating the host between epidermal cells (Ec). Scale bar=20 µm. (C) Longitudinal section of the body of an adult *M. persicae* aphid feeding on an Arabidopsis leaf. Scale bar=100 µm. (D) Close up of (C) showing a section of the stylet penetrating intercellularly. Scale bar=20 µm. (E) and (F) Immunolabeling of the slices shown in bright field in (C) and (D), respectively. The monoclonal antibody LM19 was used to target de-methylesterified HG (green) (Verhertbruggen *et al.*, 2009). The images reveal a zone of HG de-methylesterification (dme-HG) surrounding the stylet pathway. Calcofluor white was applied to reveal cell walls (magenta). Scale bar=50 µm (E) and 20 µm (F).Images (A–F) were visualized with a Leica confocal microscope model TCS LSI.

Phytophagous insects have different strategies to extract nutrients from plants. In the case of aphids, their slender stylet, which tapers from 4.5 µm in diameter near the head to 2.7 µm near the tip (Forbes, 1969), allows them to penetrate the host between epidermal cells (Fig. 1) and probe the tissues intercellularly, through the cell walls (Tjallingii and Esch, 1993) until the vascular bundles are reached, upon which sieve elements are punctured to suck the nutrients transported by the phloem.

Stylets, the piercing–sucking mouthpart of aphids, are actually composed of a bundle of four stylets: two external stylets which contain a central nerve duct, acting as a mechanosensory element during probing, plus two inner stylets whose intercalated grooves form the food ingestion and salivary channels (Forbes, 1969; Tjallingii and Esch, 1993; Miles, 1999). During probing movements toward the phloem, aphids regularly produce and inject salivary secretions into the host, containing different factors that facilitate both feeding and infestation processes (Miles, 1999; Tjallingii, 2006; Will and Vilcinskas, 2015). For example, as the stylet probes through the apoplast, a continuous salivary sheath is formed around the stylet bundle, providing mechanical stability and sealing the stylet penetration site in the sieve tube (Miles, 1999; Abdellatef *et al.*, 2015). Silencing the expression of the salivary sheath protein (SHP) in the pea aphid *Acyrthosiphon pisum* significantly reduced the duration of phloem sap ingestion, lowering the reproduction rate (Will and Vilcinskas, 2015). Also, the study of Will *et al.* (2007) demonstrated that aphid saliva prevents the plugging mechanism of sieve elements; the stylet puncture site in the sieve tube is not plugged, leading to sustained phloem ingestion. Moreover, proteomic studies have shown that the repertory of enzymes and proteins present in salivary secretions varies between aphid species and even biotypes, suggesting that they influence the infestation behavior and hence host colonization compatibility and performance (Campbell and Dreyer, 1985; Nicholson *et al.*, 2012; Thorpe *et al.*, 2016). Within this repertory, different cell wall-modifying factors and enzymes have been identified, such as a cellulose-hydrolyzing factor (Adams and Drew, 1965), and pectin methylesterase (PME) and polygalacturonase (PG) activity (Dreyer and Campbell, 1984; Ma *et al.*, 1990; Cherqui and Tjallingii, 2000). For a more thorough understanding, here we review the impact and influence of the feeding strategy of aphids and their CWMEs on the different domains of the plant cell wall, namely pectins, hemicellulose, cellulose, and callose.

## Pectic domain

The term ‘pectin’ refers to the group of polymers present in plant cell walls which share the characteristic feature of α-d-(1→4)-linked galacturonic acid (GalA) units as part of their scaffold (Willats *et al.*, 2006; Levesque-Tremblay *et al.*, 2015). Approximately 30% of cell wall material in vascular plants corresponds to pectic polymers (Ochoa-Villareal *et al*., 2012), made up of homogalacturonan (HG), xylogalacturonan (XGA), and the two rhamnogalacturonans RG-I and RG-II (Ridley *et al.*, 2001; Ochoa-Villareal *et al*., 2012). The biosynthesis, structure, and functions of pectins have been reviewed in detail elsewhere (Ridley *et al.*, 2001; Willats *et al.*, 2006; Ochoa-Villareal *et al*., 2012; Levesque-Tremblay *et al.*, 2015). Since the literature relating pectins to a defensive role in plants has mainly focused on the HG domain, we will review the influence of this pectic polymer on plant–aphid interactions.

### Homogalacturonan and its modifying enzymes

HG is a homopolymer composed of GalA residues methylesterified at C-6 (Ridley *et al.*, 2001). HGs are synthesized in the Golgi apparatus in a highly methylesterified state; ~80% of its GalA residues are methylesterified (Ibar and Orellana, 2007). Once HGs are transported to the cell wall matrix, their methylesterification state is modified by PMEs, which remove the methylester groups (EC 3.1.1.11). In turn, these reactions of HG de-methylesterification are regulated by PME inhibitors (PMEIs) (Hothorn *et al.*, 2004; Caffall and Mohnen, 2009; Levesque-Tremblay, 2015).

Depending on the methylesterification status, HGs can be directed into different fates, such as polymer breakdown by PGs (EC 3.2.1.15) and pectate lyase enzymes (PLs; EC 4.2.2.2), causing cell wall loosening, or ionic cross-linking with other de-methylesterified HG chains through calcium bridges which generally leads to cell wall stiffening (Braccini *et al.*, 1999; Willats *et al.*, 2001; Levesque-Tremblay, 2015). Therefore, by modulating the degree of methylesterification and polymerization of HG, the mechanical properties of cell walls and the middle lamella can be regulated, allowing the control of plant developmental processes such as cell expansion and plant growth (Peaucelle *et al.*, 2008; Levesque-Tremblay, 2015).

### Plant defense mechanisms related to homogalacturonan and its modifying enzymes

The evidence relating HGs to the defense response of plants includes a broad spectrum of pathogen-resistant or -susceptible phenotypes created by altering the expression levels of HG-modifying enzymes in different plant species (Cantu *et al.*, 2008). For example, the heterologous expression of a pear fruit polygalacturonase inhibitor (PGIP) in tomato significantly reduced the infection symptoms of the necrotrophic fungus *Botrytis cinerea* (Powell *et al.*, 2000). The same pear PGIP overexpressed in *Vitis vinifera* plants led to a significant decrease in the infection symptoms caused by *Xyllela fastidiosa* and *B. cinerea* (Agüero *et al.*, 2005). *Arabidopsis thaliana* plants overexpressing *Capsicum annuum* PME1 (*CaPMEI1*) showed increased resistance to *Pseudomonas syringae* pv. *tomato* (An *et al.*, 2008), whilst overexpression of *Fragaria × ananassa* PME (FaPE1) in *F. vesca* enhanced fruit resistance to *B. cinerea* (Osorio *et al.*, 2008). In addition, silencing the expression of PME1 in *Nicotiana attenuata* (*NaPME1*) leads to an increased performance (larval mass) of *Manduca sexta* larvae compared with wild-type plants (Körner *et al.*, 2009). These are just a few of many examples that highlight the influence of HG-modifying enzymes on plant defenses in response to biotic stresses.

**Fig. 2.**
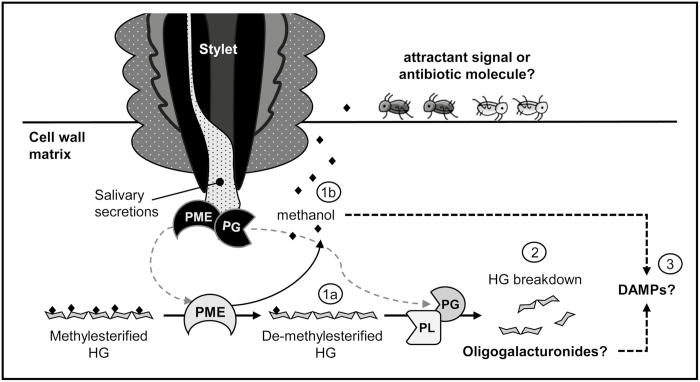
Illustrative model showing the main changes described in the HG pectic domain during aphid infestation and its hypothesized defensive role. (1a) Due to the rise in global PME activity (plant PMEs plus salivary PMEs from aphids), the abundance of de-methylesterified HG increases during aphid feeding. (1b) As a product of PME activity, methanol emissions increase, possibly acting as an attractant signal or antibiotic molecule depending on its concentration and timing. (2) Due to the increase in global PL activity and the presence of PG activity in the salivary secretion of aphids, the de-methylesterified HG chains could become depolymerized, leading to the production of OGs. (3) Both methanol and OGs produced during aphid feeding could be recognized as DAMP signals by the host plant, triggering defense responses against the attacker.

The molecular basis relating HGs to the defense mechanism of plants relies on the degradation that this homopolymer is subjected to during infections with pathogens possessing CWMEs such as PMEs, PGs, and PLs as virulence factors (Cantu *et al.*, 2008; Kubicek *et al.*, 2014; Malinovsky *et al.*, 2014). Once these pathogens enter the host, cell wall polysaccharides are degraded in order to access the cytosolic content, but also to be used as a direct carbon source by the attacker. During this process, HGs are de-methylesterified by an increase in PME activity and then depolymerized by the action of PGs and/or PLs. These activities give rise to the production of HG oligomers, named oligogalacturonides (OGs), which are biologically active molecules that the infected plant recognizes as damage-associated molecular patterns (DAMPs) (Côté *et al.*, 1998; Kubicek *et al.*, 2014; Lionetti *et al.*, 2017; Fig. 2). Once produced, OGs are sensed by the extracellular pectin-binding domain of the wall-associated kinase (WAK) receptors, triggering a defense response through the mitogen-activated protein kinase (MAPK) signaling cascade (Decreux *et al.*, 2006; De Lorenzo *et al.*, 2011; Kohorn, 2016; Bacete *et al.*, 2018).

OGs elicit different defense responses; for example, treatments of grapevine leaves with OGs reduced the susceptibility to *B. cinerea*, decreasing the lesion area of this necrotrophic fungus by up to 65% (Aziz *et al.*, 2004). Short OGs (DP=3) induced an up-regulation of pathogen-related genes and decreased susceptibility to the necrotrophic bacteria *Pectobacterium carotovorum* in Arabidopsis seedlings (Davidsson *et al.*, 2017). Moreover, altering the expression of the OG receptors (WAKs) leads to dramatic changes in pathogen resistance phenotypes in plants. For example, by challenging Arabidopsis mutants for the WAK-like receptor (WAKL; At1g67000) with different pathogens, Sopeña-Torres *et al.* (2018) found that these mutant plants were significantly more susceptible to pathogen infection since they showed increased growth of the fungus *Plectosphaerella cucumerina*, higher spore formation of *Hyaloperonospora arabidopsidis*, and a higher count of colony-forming units of *Pseudomonas syringae* pv. tomato DC3000 with respect to wild-type plants. In addition, rice plants overexpressing the *OsWAK25* gene possess an increased resistance to hemibiotrophic pathogens compared with wild-type plants, since they showed smaller lesions when challenged with *Xanthamonas oryae* pv. *oryzae* and a smaller number of sporulating lesions after infection with *Magnaporthe oryzae*. On the other hand, an opposite effect was observed when these *OsWAK25*-overexpressing lines were challenged with necrotrophic pathogens, since the lesion sizes were significantly larger after infections with *Cochliobolus miyabeanus* and *Rhizoctonia solani* compared with the wild-type genotype (Harkenrider *et al.*, 2016), thus indicating the HG pectic domain and its signaling through WAK receptors as a key element for the plant defense mechanism. Synthesis, signaling, and related defense mechanisms of OGs have been extensively reviewed elsewhere (Ochoa-Villareal *et al*., 2012; Vallarino and Osorio, 2012; Ferrari *et al.*, 2013; Kohorn, 2016; Bacete *et al.*, 2018).

### Defensive role of homogalacturonan during plant–aphid interactions

#### The attacker perspective

Although the evidence relating to HG metabolism during aphid feeding is limited, some authors propose that the presence of HG-modifying enzymes such as PME and PG in the salivary secretions of aphids could facilitate stylet penetration (McAllan and Adams, 1961; Dreyer and Campbell, 1987; Ma *et al.*, 1990). This hypothesis was first presented by McAllan and Adams (1961) where the authors correlated the occurrence of pectin hydrolytic activity of aphid saliva with the probing/penetration patterns of stylets (i.e. intercellularly or directly through the cells) in different species of aphids. They found that all the aphid species sampled with pectinase activity were able to penetrate the host intracellularly as well as through the cells. Interestingly, aphid species lacking pectin hydrolytic activity were still able to penetrate the pectic middle lamella (intercellularly). Thus, these results led the authors to suggest that pectinase activity aids intercellular penetration, but pectin hydrolysis is not necessary for aphids that penetrate directly through the cells. The study of Campbell and Dreyer (1984) also pointed to pectin and its modifying enzymes as a central element for host resistance/susceptibility. The authors compared two sorghum varieties, one of them resistant and the other susceptible to the aphid *Schizaphis graminum.* The resistant variety possessed a higher degree of methylesterification of pectin compared with the susceptible one. Interestingly, the resistant sorghum variety became susceptible when it was challenged with a new biotype of *S. graminum* that had higher basal PME activity compared with the initial biotype. These results suggest that both the degree of pectin methylesterification of the host and PME activity levels of salivary secretions could influence the colonization performance of aphids. On the other hand, some authors propose that stylet movement towards the phloem might well be explained mechanically, rather than by hydrolytic enzyme reactions (Tjallingii and Esch, 1993), arguing that ‘stylet penetration seems to go faster than the enzyme activity would allow’ (Cherqui and Tjallingii, 2000). Whether or not pectin-modifying enzymes aid intercellular stylet probing is a question that still remains elusive due to the difficulties in separately studying the mechanical and enzymatic factors of stylet penetration. For example, there are no techniques available that allow researchers to mimic the anatomy and mechanistics of stylet movements through the extracellular matrix, and hence the mechanical aspects of stylet probing cannot be assayed in isolation, independently from the enzymatic factors of salivary secretions. However, whole-genome sequencing of aphid species (International Aphid Genomics Consortium, 2010; Wenger *et al.*, 2017; Chen *et al.*, 2019) plus the molecular tools available for insect transgenesis or genome editing (Scolari *et al.*, 2011; Gantz *et al.*, 2015) could allow the knock out/down of expression of aphid CWMEs. The subsequent influence over the feeding and infestation performance could then be evaluated, permitting new insights into studying these six-decades-old questions. Moreover, a novel technique used by Will and Vilcinskas (2015) could be applied to resolve these doubts. These authors employed an artificial diet where aphids were reared to deliver an interfering RNA designed to silence the expression of the structural protein of the salivary sheath (SHP). This strategy resulted in the incapacity of aphids to correctly form this structure, leading to lower feeding and reproduction performance. They thus demonstrated the efficiency of this technique to silence the expression of aphid genes and evaluate their influence on plant–aphid interactions.

#### The host plant perspective

The role of the salivary CWMEs for the aphid pathogens is thus still unclear. Nevertheless, and in order to obtain a clearer and more holistic perspective, it is necessary to study and understand the protective function of plant CWMEs during aphid infestation. De Vos *et al.* (2005) investigated the transcriptional profile of Arabidopsis plants challenged by different attackers, such as necrotrophic and biotrophic pathogens, a chewing caterpillar, thrips, and the generalist aphid *Myzus persicae.* They found that plants up-regulated the expression of attacker-specific genes, that in the case of *M. persicae* corresponded to *PECTIN METHYLESTERASE INHIBITOR 13* (*AtPMEI13*; AT5G62360). By exploiting this valuable information, Silva-Sanzana *et al.* (2019) characterized the role of PMEI13 during aphid infestation. These authors showed that aphids significantly preferred *pmei13* mutants as host compared with wild-type genotypes. Moreover, *pmei13* mutants showed an increased susceptibility in terms of phloem accessibility and nutrient drainage, since aphids reached the phloem significantly faster on mutant plants concomitant with longer phloem sap ingestions compared with the wild-type genotypes, revealing that PMEI13 is a critical factor involved in plant resistance against aphids. Moreover, the study also showed that *M. persicae* infestation induced a significant increase in total PME and PL enzymatic activities along with an increase in the abundance of de-methylesterified HGs and methanol emissions. These results are particularly interesting considering that an increase in total PME and PL activities could lead to the production of OGs; therefore, the modification of HGs observed in early aphid infestations could correspond to a plant defense mechanism against aphids. Indeed, this hypothesis was previously proposed by Will and van Bel (2008) who speculated that aphid CWMEs present in the salivary section may lead to the production of OGs which elicit local defense responses such as the production of callose deposits and hydrogen peroxide. The model proposed by Will and van Bel (2008) also mentioned that during aphid feeding ‘the diffusion range of OGs may be restricted to the close vicinity of the stylet sheath, leading to an enhanced regional defense with a limited sphere of action’, which is consistent with the local increase in the abundance of de-methyleserified HGs described in Silva-Sanzana *et al.* (2019) where HG modifications were consistently found close to the stylet probing sites (Fig. 1). As mentioned before, OGs have been described as a critical defensive element during pathogen infection. However, their potential role during plant–aphid interactions is still just a hypothesis.

In addition to the production of OGs during pectin degradation, the de-methylesterification of HGs caused by the action of PME also leads to the production of methanol (EC 3.1.1.11; Fig. 2). This volatile molecule is emitted in large amounts by plants during caterpillar feeding and mechanical wounding (Peñuelas *et al.*, 2005; Von Dahl *et al.*, 2006; Dorokhov *et al.*, 2012). It has also been demonstrated that methanol acts as a signal for plant–plant communication since the methanol emitted by mechanically wounded plants enhances the resistance to bacteria in neighboring methanol-receiver plants (Dorokhov *et al.*, 2012). Additionally, Hann *et al.* (2014) demonstrated that methanol acts as an effective defense-eliciting DAMP in monocot grasses and, in the case of dicot plants, can modulate the defense signaling triggered by DAMPs and microbe-associated molecular patterns (MAMPs). Considering that early aphid feeding induced a rise in PME activity and methanol emissions (Silva-Sanzana *et al.*, 2019), a new question arises concerning the role of methanol during plant–aphid interaction. Results from Dixit *et al.* (2013) showed that tobacco plants overexpressing an Arabidopsis PME and possessing 16-fold higher methanol emissions than wild-type plants were significantly more resistant to *M. persicae*, since the population of aphids that fed for 6 d on transgenic lines was reduced by up to 99%.On the other hand, in a dual free choice assay, Silva-Sanzana *et al.* (2019) showed that Arabidopsis plants infiltrated with a methanol solution were significantly more preferred by *M. persicae* compared with control plants. Therefore, both studies point to methanol as a critical element influencing the host preference and colonization performance of aphids, and it is logical to postulate that the timing and concentration of the methanol emission could lead to different responses (positive or negative) on aphid behavior and plant responses in a species-specific manner.

## Hemicellulose domain

Hemicelluloses are synthesized in the Golgi apparatus by the action of glycosyltransferases. Once in the apoplast, these polymers tether cellulose microfibrils. The interaction with cellulose through hydrogen bonding and/or van der Waals forces regulates the elasticity and strength of cell walls and hence its expansion features (Bergander and Salmén, 2002; Morris *et al.*, 2004; Gu and Catchmark, 2013). The term hemicellulose refers to the group of polysaccharides possessing β-d-(1→4)-linked backbones of glucose, mannose, or xylose in an equatorial configuration at the C1 and C4 residues. Hemicellulose structure, synthesis, and biological functions have been reviewed in detail elsewhere (Scheller and Ulvskov, 2010; Ochoa-Villareal *et al*., 2012).

Of hemicelluloses, xyloglucan has been found in every plant species analyzed and is the most abundant in dicotyledonous primary cell walls (Scheller and Ulvskov, 2010). Two different mechanisms modify xyloglucan chains by the action of the cell wall-localized xyloglucan endotransglucosylase/hydrolases (XTHs) which could (i) graft xyloglucan chains to other oligosaccharides or other available xyloglucan chains (XET, endotransglucosylase activity; EC 2.4.1.207) or (ii) hydrolyze xyloglucan chains (XEH, hydrolase activity; EC 3.2.1.151) (Maris *et al*., 2011).

As mentioned before, upon pathogen infection, for successful host colonization, cell wall polymers are a direct target for degradation. Indeed, xyloglucan is an essential barrier torn down by fungi since it is proposed that the decrease in XET activity observed upon tomato fruit infection by *Penicillium expansum* could be a sabotage mechanism of this pathogenic fungus to increase tissue maceration and hence favor host colonization (Miedes and Lorences, 2007). Moreover, from the host perspective and similarly to the defense-eliciting activity of OGs, it has been demonstrated that treatments with xyloglucan oligomers in Arabidopsis and *V. vinifera* lead to increased resistance to *B. cinerea* and to the biotrophic oomycete *Hyaloperonospora arabidopsidis*, achieved by the activation of a MAPK signaling cascade (Claverie *et al.*, 2018). These studies demonstrate that xyloglucan possesses a central role during plant–pathogen interactions.

However, few studies have investigated the influence of hemicelluloses and their modifying enzymes over plant–aphid interactions. Divol *et al.* (2005) showed that during *M. persicae* infestation of celery (*Apium graveolens*), the transcript abundance of *ENDOTRANSGLUCOSYLASE/HYDROLASE 1* (*AgXTH1*) rises significantly in systemic phloem tissue. Then, in a subsequent study, the influence of Arabidopsis *XTH33* (homologous to *AgXTH1*) on aphid infestation behavior was evaluated, revealing that aphids significantly preferred to settle on *xth33* mutants compared with the wild-type genotype (Divol *et al.*, 2007). In addition, Rasool *et al.* (2017) showed that *M. persicae* infestation induces a drastic reduction in the abundance of galactosylated xyloglucans in tobacco plants. Therefore, both results indicate an influence of xyloglucan and its modifying enzymes over aphid–plant interactions.

## Cellulose domain

Cellulose is built up of β-d-(1→4) glucan chains synthesized by CELLULOSE SYNTHASE A (CESA) protein complexes at the plasma membrane. As soon as the chains are synthesized, they gather together via intermolecular hydrogen bonding, leading to the formation of cellulose microfibrils (Somerville, 2006; McFarlane *et al.*, 2014). These microfibrils wrap the plant cells in overlapping layers, providing a rigid network controlling turgor pressure, and hence cell expansion and the upright growth habit of terrestrial plants (Abe *et al.*, 1997; Somerville, 2006; Chan, 2012).

Regarding the defensive role of this β-d-(1→4) glucan polymer, cellulose participates in the basal responses during infection by forming papillae structures along with callose to limit the penetration and spread of the pathogen (detailed below in the callose section). Additionally, like the pectic oligomers (OGs), cellulose dimers (cellobiose) act as DAMP signals with defense-eliciting activity through the activation of MAPK signaling cascades, leading to an up-regulation of salicylate-, jasmonate-, and ethylene-related genes in Arabidopsis (de Azevedo Souza *et al.*, 2017).

Regarding the influence of cellulose on plant–aphid interactions, just one study has, in part, addressed this topic. By using *cev1* plants of Arabidopsis, mutated in the cellulose synthase gene *CESA3*, Ellis *et al.* (2002) showed that the colonization performance of *M. persicae* is significantly reduced in mutants compared with wild-type plants, by using a non-choice assay where aphids are forced to colonize a particular genotype. The authors concluded that these results are due to the fact that *cev1* plants have constitutively active jasmonate and ethylene signaling pathways (Ellis and Turner, 2001; Ellis *et al.*, 2002). Indeed, *cev1* mutants also showed increased resistance to the biotrophic fungus *Erysiphe cichoracearum* and the hemibiotrophic bacterium *P. syringae* pv. *maculicola* (Ellis *et al.*, 2002). Moreover, more than a half a century ago, Adams and Drew (1965) described the presence of cellulose-hydrolyzing activity in the salivary secretions of several aphid species by *in vitro* assays, although, at present, no studies have yet addressed their influence over the feeding and infestation mechanisms of these phloem-feeding insects.

## Callose

Callose is a β-d-(1→3) glucan linear homopolymer of glucose residues with less frequent β-d-(1→6) glucan branches (Stone, 2009; Nedukha, 2015). The synthesis of this polymer occurs in a calcium-dependent manner (Kauss, 1985; Thonat *et al.*, 1993) at the plasma membrane of plant cells and, in the case of Arabidopsis, is carried out by a family of enzymes composed of 12 members named callose synthase (CalS) (Shi *et al.*, 2016). Callose has been found to participate in different molecular and physiological processes of plants such as the formation of sieve plates (Levy and Epel, 2009) and the cell plate during late cytokinesis (Verma, 2001). Callose also controls symplastic trafficking by regulating homeostasis of plasmodesmata (De Storme and Geelen, 2014; Wu *et al.*, 2018), provides mechanical resistance to tension and compression during pollen tube development (Parre and Geitmann, 2005), and prevents sieve element leakage after mechanical wounding or heat shock stress (Thonat *et al.*, 1993; Furch *et al.*, 2007).

In the context of biotic stress, it has been described that during fungal infections, callose along with cellulose forms amorphous clogging deposits (papillae) at the sites where pathogen CWMEs have degraded plant cell walls, and hence limits the penetration and spread of the attacker (Voigt and Somerville, 2009; Ellinger *et al.*, 2013; Chowdhury *et al.*, 2014). For example, the work of Ellinger *et al.* (2013) showed that the overexpression of the *POWDERY MILDEW RESISTANT 4* gene (*PMR4*; encoding a pathogen-induced callose synthase) in Arabidopsis increases callose deposition at the early stage of infection (6 h) compared with wild-type plants, leading to a phenotype described as complete resistance to penetration, upon infections with two powdery mildew fungi, virulent *Golovinomyces cichoracearum* and non-virulent *Blumeria graminis*. This study revealed that callose–cellulose papillae could abolish the infection process, regardless of the compatibility of the pathosystem. Moreover, in a later study, by using localization microscopy, Eggert *et al.* (2014) demonstrated that PMR4-overexpressing Arabidopsis plants not only synthesize more extensive local deposits of callose, but callose also spreads into the adjacent cellulose fibrils close to the infection site, suggesting that this tight cellulose–callose network prevents the action of the attacker CWMEs and hence their penetration of cell walls, highlighting a previously unknown defense mechanism related to callose.

After physical injuries, sieve tube occlusion is needed to prevent the phloem sap leakage. To this end, plants have evolved protein-related mechanisms of sieve tube clogging, which vary depending on the plant family studied, for example the forisome system in the case of the Fabaceae family (Will *et al.*, 2009). Alternatively, callose deposition seems to be a universal mechanism of phloem element clogging after stylet disruption by aphids.

It is thought that callose deposits upon aphid infestation could occur as a downstream defense mechanism related to oligogalacturonides produced by the pectin-degrading enzymes of salivary secretions (Will and van Bel, 2008). This hypothesis is based in the evidence shown in Denoux *et al.* (2008) where Arabidopsis plants infiltrated with oligogalacturonide solution accumulate callose deposits.

The leading role of this β-glucan polymer is related to the plugging of the pierced sieve elements and the punctured non-phloematic cells along the route of intercellular stylet probing. For example, in wheat leaves, a significant accumulation of callose deposits was found in sieve plates, plasmodesmatal pores of companion cells, and stylet tracks upon infestation by the Russian wheat aphid, *Diuraphis noxia* (Botha *et al.*, 2004). Moreover, infestation of barley with aphids *Rhopalosiphum padi*, *M. persicae*, *M. cerasi*, or *Diuraphis noxia* resulted in callose deposition in epidermal cells, sieve plate pores, and plasmodesmatal pores (Saheed *et al.*, 2009; Escudero-Martinez *et al.*, 2017). This evidence is especially interesting since depending on their feeding habit, aphids can be classified as generalists or specialists (Schoonhoven *et al.*, 2005). Specialist aphids have the capacity to colonize a few, closely related species of plants for which its whole physiology and behavior have evolved to specifically exploit such hosts by overcoming their defense mechanisms, while generalists have the capacity to take advantage of a greater number of species, even though colonization performance is less efficient than that of specialists (Bernays and Funk, 1999; Stilmant *et al.*, 2008). Therefore, in this case, where the aphid species analyzed possess different degrees of specialization to colonize barley, callose depositions appear to be a universal defense factor within basal host resistance, independent of the feeding habit of aphids.

From the attacker’s perspective, the callose plugging of the sieve elements punctured by stylets represents a defense mechanism that hinders the sap ingestion. However, that the calcium-chelating activity of the watery salivary secreted into the sieve tube before sap ingestion (Tjallingii and Esch, 1993) is described to suppress the forisome clogging system *in vitro* (Will *et al.*, 2007). Some authors suggest that this sabotage mechanism could also impede the calcium-dependent synthesis of callose deposits (van Bel and Will, 2016). In addition, van Bel and Will (2016) also propose the idea that aphids could overcome callose plug synthesis by an up-regulation of host β-1,3-glucanases induced by salivary effectors secreted into the host. This idea is supported by the work of Mehrabi *et al.* (2016), where barley genotypes susceptible to *R. padi* showed higher expression levels of β-1,3-glucanases upon aphid infestation compared with the resistant genotypes, thus suggesting that these callose-degrading enzymes correspond to susceptibility factors upon aphid infestation. Although the putative salivary effectors proposed to manipulate the expression of host β-1,3-glucanases remain unidentified, the results shown in Elzinga *et al.* (2014) could provide the evidence to support this hypothesis. This study revealed that Arabidopsis plants expressing the *M. persicae* salivary protein Mp55 showed lower accumulation of callose deposits upon aphid infestation. Also, the work of Naessens *et al.* (2015) showed that the number of callose deposits induced after cryptogein (a microbe-associated molecular pattern) treatments in *Nicotiana benthamiana* leaves was significantly reduced by transient expression of the *M. persicae* salivary factor, MIF1, therefore demonstrating that the salivary protein of aphids manipulates callose synthesis to overcome this sieve tube clogging mechanism.

## Unanswered questions

- Could the OG–WAK–MAPK complex be a convergence node of plant immunity to sense and trigger a cross-kingdom defense mechanism, ranging from fungi and bacteria to aphids?
- Considering that aphids ‘drink’ from xylem cells and thus are mechanistically unable to penetrate these thick, lignin-reinforced cell walls, could pectin rheology really represent a significant physical obstacle for stylet penetration?
- Aphids are attracted but also killed by methanol emissions of plants depending on its concentration; thus, is there is a threshold of methanol emission to turn it from an attractant molecule to an antibiotic compound? Alternatively, could these experimental approaches induce an as yet unknown side effect of methanol on host plants, hence altering aphid behavior?
- MAPK phosphorylation seems to be a common feature after sensing of cell wall-derived DAMPs. However, how similar are the responses downstream of DAMP perception upon bacterial, fungal, and aphid attack?

## Final remarks and future challenges

Although the detailed defensive mechanism of HGs during plant–aphid interactions has yet to be completely described, the evidence accumulated to date points to this pectic polymer as a central element of plant defense responses against these pests distributed worldwide.

The mechanism of perception of xyloglucan and cellulose oligomers remains uncharacterized. However, the phosphorylation of MAPK upon treatments with these DAMPs suggests the participation of receptor-like kinase (RLK) members, similar to WAKs.

Colonization behavior and performance of aphids are influenced by alterations in the methanol emissions of host plants. However, a deeper understanding of the influence of this product of PME reactions is needed, since the timing and concentration of the emissions seem to produce different effects on plant–aphid interactions.

Considering the impact of cell wall polymer status and CWMEs on pathogen infections and aphid behavior, cell wall-related elements could represent an input to develop new crop traits pointing to insect/pathogen resistance.

Whether stylet penetration is only performed by mechanical forces or corresponds to a CWME-assisted process is an ongoing question that has not yet been clearly answered due to the complexity of separately analyzing both components. Nevertheless, the current availability of whole-genome sequences of different aphid species plus the accessibility to gene editing/silencing techniques represent the opportunity to elucidate the intriguing feeding strategy of aphids.

## Abbreviations

CWME: cell wall-modifying enzyme
DAMP: damage-associated molecular pattern
HG: homogalacturonan
OG: oligogalacturonide
PG: polygalacturonase
PL: pectate lyase
PME: pectin methylesterase
